# Preconception expanded carrier screening: Impact of information presented by text or video on genetic knowledge and attitudes

**DOI:** 10.1002/jgc4.1332

**Published:** 2020-09-17

**Authors:** Thirsa Conijn, Stephanie C. M. Nijmeijer, Phillis Lakeman, Lidewij Henneman, Frits A. Wijburg, Lotte Haverman

**Affiliations:** ^1^ Pediatric Metabolic Diseases Emma Children's Hospital and Amsterdam Lysosome Center “Sphinx” Amsterdam UMC University of Amsterdam Amsterdam Netherlands; ^2^ Psychosocial department Emma Children's Hospital Amsterdam UMC, University of Amsterdam Amsterdam Netherlands; ^3^ Department of Clinical Genetics Amsterdam Reproduction and Development Research Institute Amsterdam UMC University of Amsterdam Amsterdam Netherlands; ^4^ Department of Clinical Genetics Amsterdam Reproduction and Development Research Institute Amsterdam UMC Vrije Universiteit Amsterdam Amsterdam Netherlands

**Keywords:** attitudes, education, expanded carrier screening, genetic knowledge, mucopolysaccharidosis type III, population screening, text, video

## Abstract

Preconception expanded carrier screening (ECS) aims to identify couples with an increased risk of having a child with an autosomal recessive (AR) disorder before pregnancy, thereby enabling reproductive choices. Genetic knowledge and experiential knowledge both influence the uptake of ECS. As people in the general public often lack such knowledge, it is essential to provide appropriate and understandable information when offering ECS. This study investigated the effect of an educational video, compared to an educational text, on the knowledge and attitudes toward preconception ECS in the general population. Both the text and video consisted of a brief educational summary on AR inheritance and on the type of disorders included in ECS, with the progressive neurodegenerative condition mucopolysaccharidosis type III (MPS III) as an example. Participants in the reproductive age were invited in collaboration with a research agency. Respondents (*N* = 789) were offered an educational video prior to completing an online questionnaire that examined genetic knowledge, the perceived severity of MPS III, perceived risk, and attitudes toward ECS. Outcomes were compared to reference data collected previously in which respondents had been offered an educational text (*N* = 781). We first again studied the attitudes toward ECS in a smaller educational text group (*N* = 266) in order to assess whether attitudes had changed over time due to increased media coverage on ECS, which did not reveal any significant changes. Respondents who were offered the video had a better genetic knowledge, perceived MPS III as more severe, perceived their risks higher and were more likely to participate in ECS compared to those who were offered text. Online video may well be used as supportive tool to the genetic counseling process, creating more knowledge on ECS and severe genetic disorders included in preconception screening panels.

**What is known about this topic**

Genetic knowledge and experiential knowledge on disorders included in screening panels both influence the uptake of preconception expanded carrier screening.

**What this paper adds to the topic**

The use of video for educating the general public on preconception expanded carrier screening and on the disorders included in such tests leads to increased genetic knowledge and may well be used as supportive tool to the genetic counseling process.

## 1 INTRODUCTION

Patients with autosomal recessive (AR) disorders are generally born into families with no prior family history for that specific disorder. Carrier couples are thus mostly not aware of their carrier status and increased risk of having affected offspring (Archibald et al., 2018; Ropers, 2012). Preconception expanded carrier screening (ECS) entails simultaneously testing for multiple AR disorders and provides the opportunity to identify carrier couples before pregnancy, thus allowing autonomous reproductive choices (Chokoshvili, Vears D, & Borry, 2018; Henneman et al., 2016). The number of AR disorders included in available ECS panels varies from 40 to more than 1,000 disorders (Chokoshvili, Borry, & Vears, 2017). Since 2016, universal preconception ECS panels for 50 to 70 severe AR childhood onset disorders have been made available to the general public in the Netherlands by two academic hospitals (Amsterdam University Medical Centers (Amsterdam UMC, n.d.) and the University Medical Centre Groningen (UMCG, 2018)). These ECS panels are available for couples from the general population, who do not have an increased risk of being a carrier couple based on ancestry or consanguinity, and are currently not reimbursed by Dutch health insurance. The panels comprise relatively more prevalent severe childhood onset disorders, resulting in significant physical disabilities and/or intellectual disabilities and/or severe pain and/or frequent hospital visits, for which no highly effective disease modifying treatment is available and/or a premature demise is expected (e.g*.,* Mucopolysaccharidosis type III (MPS III), Zellweger syndrome, and Tay‐Sachs disease), as advised by the European Society of Human Genetics (Henneman et al., 2016).

Recent studies showed that the intended uptake of universal ECS by the general population varies from approximately 30% in the Netherlands and Sweden (Ekstrand Ragnar, Tyden, Kihlbom, & Larsson, 2016; Nijmeijer et al., 2019; Plantinga et al., 2016) to as high as 68% in Western Australia (Ong et al., 2018). According the Health Belief Model, people's perception about the perceived severity of an illness, the perceived risk for a disease, and the perceived benefits or barriers influence the engagement in (preventive) health related behavior. Modifying variables, such as knowledge, can affect those perceptions (Rosenstock, 1974). Several studies indeed showed that both genetic knowledge (e.g*.,* knowledge on genetic risks and AR inheritance patterns) and experiential knowledge on the potential impact of a severe genetic disease are important factors determining a person's intention whether or not to participate in ECS programs (Boardman, Young, Warren, & Griffiths, 2018; Chen & Goodson, 2007; Holtkamp et al., 2017; Ioannou et al., 2014; Metcalfe et al., 2017; Ong et al., 2018). In the general population, there is a low level of genetic knowledge on ECS leading to misconceptions about carrier screening, for example, not recognizing that ECS can be relevant especially in the absence of a family history of a genetic disease (Henneman, Timmermans, & van der Wal, 2004; Lanie et al., 2004; McClaren, Delatycki, Collins, Metcalfe, & Aitken, 2007; Nijmeijer et al., 2019). Moreover, experiential knowledge on the nature of the diseases generally included in ECS panels is low in the general public, as most severe AR diseases are rare (Ioannou et al., 2014; Nijmeijer et al., 2019). In populations with a relatively high risk of AR disorders, such as the Ashkenazi Jewish population and genetically isolated populations, people are much more familiar with AR disorders and carrier screening (Holtkamp et al., 2017). Interestingly, studies investigating the attitudes toward preconception ECS in specific populations with experiential knowledge of a disorder included in screening panels, such as family members of cystic fibrosis (CF) or MPS III patients, reported higher percentages (up to 80%) in favor of ECS for that specific disorder (Bailey, Bishop, Raspa, & Skinner, 2012; Boardman, Hale, Gohel, & Young, 2019; Janssens et al., 2016) or for extended screening panels (Nijmeijer et al., 2020). These findings underscore the impact of personal experience with severe genetic disorders on screening attitudes.

The discrepancy in attitudes toward ECS between the general public and people with genetic and/or experiential knowledge emphasizes the importance of providing understandable and appropriate information to ensure informed decision‐making in the general public when offering ECS. This may be achieved by educational texts, leaflets, face‐to‐face conversations, or videos. The latter offers the opportunity to visualize difficult concepts and may portray real‐life situations, also to people with limited literacy (Ferguson, 2012). Several studies indeed showed that the use of video can help to increase knowledge and informed decision‐making on certain health topics (e.g., antenatal Down Syndrome screening) (Bjorklund, Marsk, Levin, & Ohman, 2012; Hardy, Kener, & Grinzaid, 2018; Schnellinger et al., 2010; Temme et al., 2015).

With this study, we aim to assess the effect of a short educational video compared to text on knowledge and attitudes toward preconception ECS in the general public. We took specific care to only use non‐directive and objective information about ECS in the video as we did not want participants to interpret the video as a recommendation to opt for ECS.

## 2 METHODS

### 2.1 Participants and procedures

Respondents in the reproductive age (18–45 years) were recruited online in October 2018 by TNS Kantar, a Dutch research agency, that provide access to a panel of more than 200,000 individuals who are willing to participate in research in exchange of a small reimbursement. The sample was stratified from their database based on the key demographics gender, socio‐economic status, educational level, and regional area to guarantee a representative sample of the Dutch population. Informed consent was obtained from all respondents. The Medical Ethics Committee of the Amsterdam UMC, the Netherlands, stated that Medical Research Involving Human Subject Act (WMO) does not apply to this study as it concerns an anonymous questionnaire study, and therefore, formal ethical approval was not necessary.

The recruiting procedure was identical to the procedure used in a previous study on the attitudes toward preconception ECS in the general Dutch population (Nijmeijer et al., 2019).

In total, 789 participants (1,745 invited, response rate 45.2%) were offered an educational video (video group) presenting information on ECS and MPS III based on the educational text offered in the study of Nijmeijer et al. (2019). The data from the 781 respondents collected in that study were used as reference data (text group). Since 2 years had elapsed between the data collection of the video group (2018) and the text group (2016), a group of 266 participants were additionally recruited in the current study (521 invited, response rate 51.1%) and offered the same text for information as used in the Nijmeijer et al. (2019) study in order to assess whether time had influenced the attitudes toward ECS possibly as the result of increased media coverage on ECS.

### 2.2 Provided information

The educational text, video, and questionnaire were all in Dutch. Supplementary materials for this publication were translated into English.

#### 2.2.1 Educational text

A full description of the original educational text as used in the study by Nijmeijer et al. (2019) can be found in Appendix S1. It concerns a brief online written educational summary on AR inheritance, the ECS test, reproductive options, and a description of the nature and course of MPS III (Sanfilippo disease) to illustrate the type of disorders included in the ECS test.

#### 2.2.2 **Educational** video

The video was designed and produced in collaboration with an organization specialized in educational videos in the field of medicine (Artsen voor Kinderen; Doctors for Children). The script of the educational text was used as frame from which the video was designed. We used informative animations with a voice over to visualize the information on AR inheritance and the ECS test. The general concept of ECS, reproductive options, and the nature and course of MPS III as an example of the type of disorders included in ECS were explained by a medical doctor, supported by visual bullets and animations. MPS III was illustrated by video‐recording of two MPS III patients in their home environment (e.g., images of patients walking with their parents or sitting in a wheelchair). In addition, extra factual information about the general risk of being a carrier couple (1 in 150) and the general risk of having an affected child with one of the AR disorders included in the ECS test (1 in 600) had been added. The video lasted 6:27 minutes (see https://youtu.be/V9FKDNF_‐tI, including English subtitles).

### 2.3 Outcome measures

#### 2.3.1 Sociodemographic characteristics

The sociodemographic characteristics age, gender, educational level, considering a (future) pregnancy, marital status, and religion were collected. Additionally, three multiple choice questions assessed familiarity with carrier screening, with hereditary diseases and previous experience with genetic carrier testing.

#### 2.3.2 Genetic knowledge

A brief genetic knowledge test was used to assess whether the information in the educational text or video was correctly understood. The genetic knowledge test consisted of seven items (e.g., ‘if only *one* partner is carrier for one of the diseases, couples have an increased risk to have a child with that disease’) and was answered on a three point scale (1 = *correct*, 2 = *incorrect*, and 3 = I *do not know*). The total score ranges from 0 to 7, at which a higher score indicates a better genetic knowledge (Appendix S2).

#### 2.3.3 Perceived severity of MPS III

The perceived severity of MPS III was assessed by two statements (‘I believe MPS III is a severe disease’ and ‘I believe that the life expectancy of MPS III is very bad (severe patients often die before reaching adulthood)’) and answered on a five‐point Likert scale (1 = *totally disagree* to 5 = *totally agree*) (Appendix S2).

#### 2.3.4 Perceived risk

Three questions about the perceived risk of being a carrier (‘How high do you consider a chance of 1:6 of being a carrier of a severe, hereditary disease?’, ‘How high do you consider a chance of 1:150 that both partners are carrier of the same severe hereditary disease?’, and ‘How high do you consider the chance of 1:600 per pregnancy of having a child with a severe, hereditary disease?’) were assessed and answered on a five‐point Likert scale (1 = *very low* to 5 = *very high*) (Appendix S2).

Answers on these three domains were also collected in the study by Nijmeijer et al. (2019) (text group), but were not reported in that publication. We now used these outcomes to examine the effect of the educational video in comparison to text on these domains.

#### 2.3.5 Attitudes toward ECS

In the current study, the following domains, also assessed by Nijmeijer et al. (2019) in their ‘text only’ study, were studied (Appendix S2):

I Intention to participate in ECS, which was measured multiple choice (1 = *definitely,* 2 = *probably*, 3 = *I do not know*, 4 = *probably not*, 5 = *definitely not*, 6 = *I already had a carrier test*).
II Level of agreement on feelings toward ECS in general and personally considering ECS, which was measured using a semantic differential five‐point scale with seven adjective word pairs: *negative–positive, undesirable–desirable, frightening–non‐frightening, unwise–wise, non‐reassuring–reassuring, unethical–ethical, illogical‐logical*.
III Perceived benefits of (A) and barriers against ECS (B), and freedom‐of‐choice statements (C), measured by using a five‐point Likert scale (1 = *totally disagree to* 5 = *totally agree*).
IV Most important reasons in favor of ECS and against ECS, measured by choosing a maximum of two arguments out of a list of arguments.
V Perceived personal consequences of ECS: Considerations regarding test results of ECS (A), Perceived consequences as a carrier (B), and Perceived reproductive choices as a carrier couple (C), measured by using a five‐point Likert scale (1 = *totally disagree to* 5 = *totally agree*).

### 2.4 Statistical analyses

Descriptive statistics were performed to describe the sociodemographic characteristics of all groups. Sociodemographic characteristics of the groups were compared by using independent sample t tests for continues data and chi‐square tests for categorical data. The items of the genetic knowledge test were transformed to binary coded items (0 = *incorrect answer/I do not know*, 1 = *correct answer*) and computed to a total sum score (range 0–7). All items containing five‐point scales were transformed to binary coded items (0 = *(totally) disagree/not disagree/not agree* or *(very) low/not low and not high,* 1 = *agree/totally agree* or *(very)high*). The word pairs (domain II) were compromised into three answer categories: 1 = *(totally) disagree*, 2 = *do not disagree/do not agree*, and 3 = *(totally) agree*. First, it was assessed whether sociodemographic characteristics, genetic knowledge, perceived severity of MPS III, perceived risk, and attitudes toward preconception ECS remained the same over time by comparing outcomes of respondents who received the text in 2016 and 2018 by using Mann–Whitney U tests and chi‐square tests. Second, answers on the genetic knowledge test, the perceived severity of MPS III, the perceived risk, and attitudes toward ECS were compared between the video group and the text group by using Mann–Whitney U tests and chi‐square tests to assess the impact of the educational video compared to text. Statistical Package for Social Sciences (SPSS) version 25 was carried out for all statistical analyses (SPSS, Inc.). *p*‐values < .05 were considered statistically significant.

## 3 RESULTS

### 3.1 Sociodemographic characteristics

No significant differences were found between respondents who received the written educational text in 2016 and in 2018 (data not shown).

The sociodemographic characteristics of the text group and video group are presented in Table 1. No significant differences were found between the groups. Respondents were in the reproductive age (18–47 years), and the majority of them had not heard of a carrier screening test before participating in this study. The minority of the respondents already had a carrier screening test (3.2% in the text group and 2.5% in the video group). All respondents were included in the analyses.

**TABLE 1:** Sociodemographic characteristics

	Text group^a^		Video group		*p*
	(*n* = 781)		(*n* = 789)		
	*n*	%	*n*	%	
Age in years; mean (SD)	31.2 (7.3)		31.4 (7.0)		.54
18–24	151	19.3	132	16.7	.14
25–34	374	47.9	413	52.3	
35–45	256	32.8	244	30.7	
46–47	0	0	2	0.3	
Female gender	379	48.5	411	52.1	.16
Educational level^b^					.55
Low	164	21.1	158	20.0	
Intermediate	356	45.7	348	44.1	
High	259	33.2	283	35.9	
Religious beliefs					.36
No	341	43.7	362	45.9	
Yes	418	53.5	398	50.4	
I do not want to say	22	2.8	29	3.7	
Marital status					.58
Single	198	25.4	205	26.0	
In a relationship/married	583	74.6	583	73.9	
Other	0	0.0	1	0.1	
Considering a (future) pregnancy					.40
No	461	59.0	449	56.9	
Yes^c^	320	41.0	340	43.1	
Currently pregnant (partner or self)	20	3.0	36	4.8	
Do you know someone (or have you known someone) with a hereditary disease?					.36
No	561	71.8	195	73.3	
Yes	220	28.2	71	26.7	
Have you ever heard of a carrier test before this questionnaire?					.34
No	604	77.3	550	75.3	
Yes	177	22.7	239	20.3	
Have you ever taken a carrier test?					.37
No	756	96.8	769	97.5	
Yes	25	3.2	20	2.5	

^a^ Data collected in 2016 by Nijmeijer et al. (2019).

^b^ Educational level was divided into three categories according to the classification of Statistics Netherlands: low (primary education, lower vocational education, lower and middle general secondary education), intermediate (middle vocational education, higher secondary education, pre‐university education), and high (higher vocational education, university. Distribution of educational levels in the Netherlands: 30% low, 40% intermediate, and 30% high.

^c^ Considering a (future) pregnancy contained the following answers: ‘I have no children at the moment but I would like to have children’, ‘I have children and my partner and I would like to have more children’, ‘I am/my partner is currently pregnant', or ‘I would have liked to have children but I remained childless’.

### 3.2 Controlling for the influence of time

There was no significant difference in the number of respondents expressing an intention to participate in ECS between the groups who received the educational text in the current study and 2 years previously in the study by Nijmeijer et al. (2019) (35.1% and 31.0% respectively, *p* = .223). Moreover, no significant differences between both groups were found in 60 of the 62 items relating all other domains. Participants in the current text group only agreed more often on the statement ‘the carrier test can avoid suffering for future parents’ (74.1% vs. 67.2%, *p* = .04) and that MPS III has a very bad life expectancy (82.0% vs. 75.0%, *p* = .021).

### 3.3 Effect of the educational video compared to the educational text

#### 3.3.1 Genetic knowledge test

Respondents in the video group scored higher on the knowledge test (median = 6, range 0–7) compared to respondents in the text group (median = 5, range 0–7, *p* < .001).

#### 3.3.2 Perceived severity of MPS III

The majority of all respondents experienced MPS III as a severe disease. However, respondents in the video group more often agreed that MPS III is a severe disease (83.7%) compared to the text group (72.3%, *p* < .001) and more often agreed that MPS III has a very bad life expectancy (84% in video group vs. 75% in text group, *p* < .001).

#### 3.3.3 Perceived risk

Respondents in the video group and the text group equally perceived a chance of 1:6 of being a carrier of a severe hereditary disease as (very) high (70.3%). Compared to the text group, the video group more often perceived a chance of 1:150 that both partners are carrier of the same disease as (very) high (respectively, 38.5% vs. 46.6%, *p*=.001) and a chance of 1:600 of having a child with a severe hereditary disorder as (very) high (respectively, 31.4% and 38.1%, *p*=.005).

#### 3.3.4 Attitudes toward preconception ECS

I Intention to participate in ECS.

The minority of respondents stated that they would participate themselves in ECS. However, respondents in the video group reported more often that they would probably or certainly participate in preconception ECS compared to respondents in the text group (39.1% vs. 31%, *p* < .001).

II Level of agreement on feelings toward ECS in general and personally considering ECS.

**FIGURE 1:**
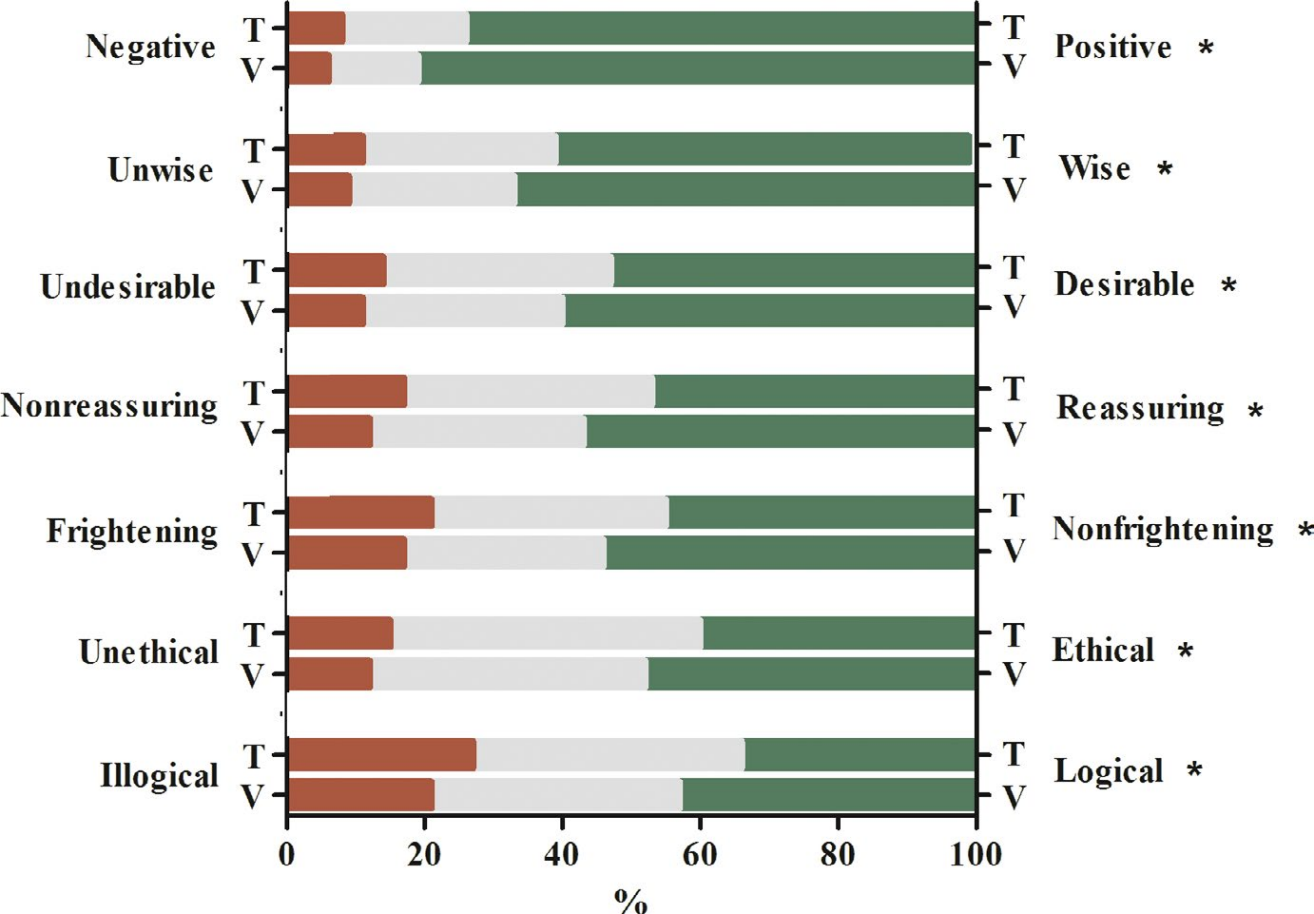
*1*Level of agreement on feelings toward ECS in general. Questionnaire domain II. The figure illustrates the level of agreement of the text group (T; *n* = 781) and the video group (V; *n* = 789) on seven feelings in response to the question: ‘That all couples considering a (future) pregnancy can take the carrier test for 50 severe hereditary disorders, I find’. The red bars represent the percentage of participants who (totally) agreed with the words on the left side of the figure. The gray bars represent the percentage of participants with a neutral opinion toward the word pairs. The green bars represent the percentage of participants who (totally) agreed with the words on the right side of the figure. *p < .05, **p < .001 by using chi‐square tests

**FIGURE 2:**
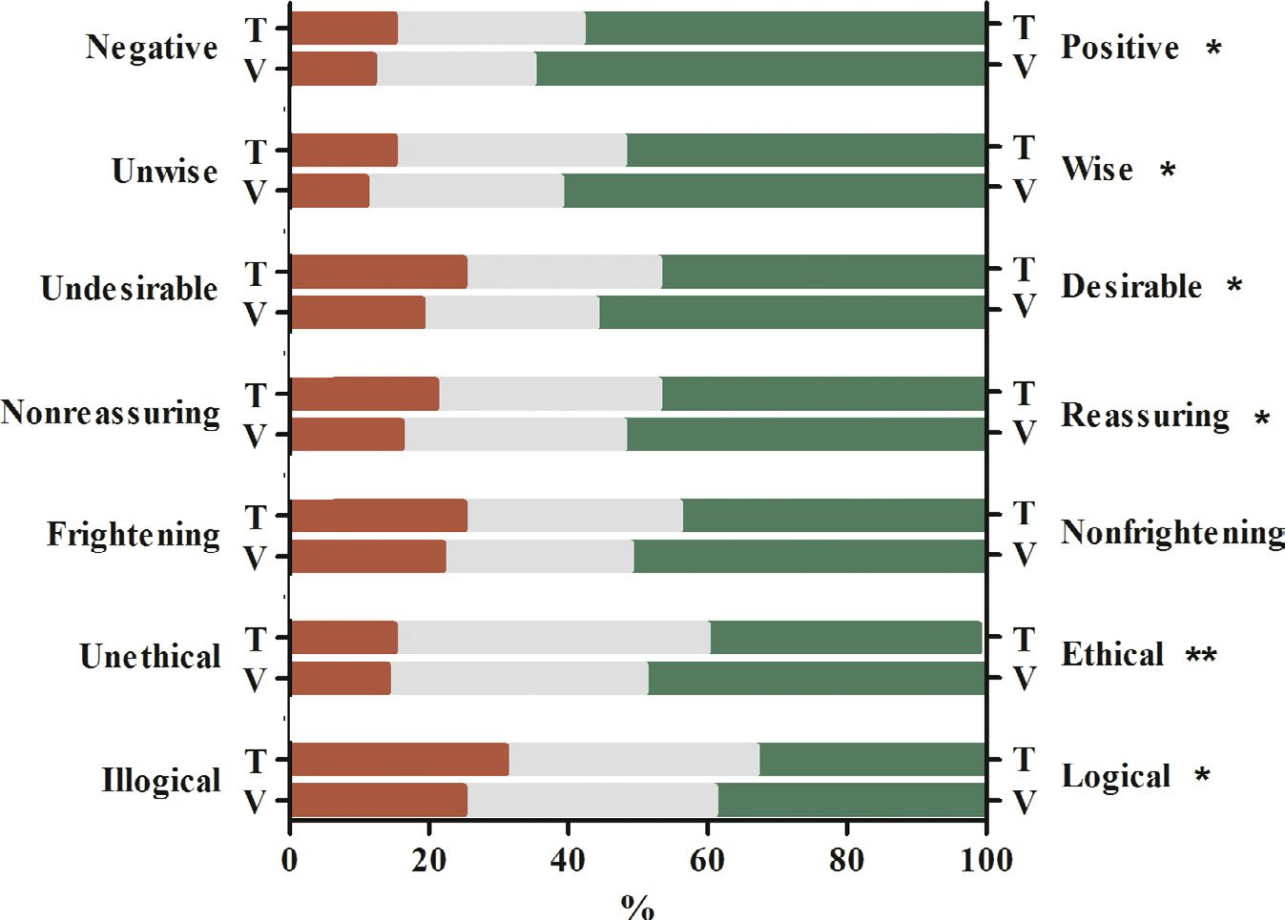
Level of agreement on feelings when personally considering ECS. Questionnaire domain II. The figure illustrates the level of agreement of the text group (T; *n* = 781) and the video group (V; *n* = 789) on seven feelings in response to the question: ‘That I personally can take the carrier test for 50 severe hereditary disorders, I find’. The red bars represent the percentage of participants who (totally) agreed with the words on the left side of the figure. The gray bars represent the percentage of participants with a neutral opinion toward the word pairs. The green bars represent the percentage of participants who (totally) agreed with the words on the right side of the figure. *p < .05, **p < .001 by using chi‐square tests

Respondents in the video group and text group significantly differed in their agreement on almost all feelings toward ECS in general (Figure 1), and when personally considering ECS (Figure 2). Agreements of respondents in the video group were more in favor of ECS compared to those in the text group.

III Perceived benefits of and barriers against ECS and freedom‐of‐choice statements.

Respondents in the video group significantly more often agreed with all statements associated with potential benefits of ECS compared to the text group (Table 2A). They also less often agreed with almost all the statements associated with potential barriers against ECS. Respondents in the video group and the text group only equally agreed with the statement that ECS may be a first step in the development of a perfect child (respectively, 31.5% and 31.6%) (Table 2B). Finally, respondents in the video group significantly more often agreed with all freedom‐of‐choice statements compared to the text group (Table 2C).

IV Most important reasons in favor of ECS and against ECS.

**TABLE 2:** Agreement with statements regarding perceived benefits of and barriers against ECS and freedom‐of‐choice statements

	Text group^a^	Video group	***p***
	(*n* = 781)	(*n* = 789)	
	%	%	
A. Perceived benefits of ECS			
The carrier test can avoid suffering for future parents	68.2	74.3	**.008**
Offering the carrier test avoids much suffering for the entire family	67.2	73.3	**.009**
The carrier test can prevent costs for the family	57.4	68.1	**.000**
The carrier test can prevent costs for the society	48.9	59.3	**.000**
The results of a carrier test can help in choosing a partner	7.30	4.80	**.039**
B. Perceived barriers against ECS			
The carrier test creates too high expectations of the birth of a healthy child	43.9	36.2	**.002**
The carrier test will be the first step in developing a perfect child	31.5	31.6	.979
Offering a carrier test leads to anxiety	39.2	30.9	**.001**
Offering the carrier test can cause people to feel forced to undergo testing	29.2	22.1	**.001**
I am afraid of discrimination by carriers (for instance, by insurance companies and the social environment)	23.4	16.0	**.000**
C. Freedom‐of‐choice statements			
The carrier test should be offered to every couple that wants to have children	54.9	65.9	**.000**
Every couple that wants to have children must take the carrier test	22.0	26.2	**.034**

Note

Questionnaire part III: agreement on statements regarding ECS.

Significant differences *p* < .05 are presented in bold.

^a^ Data collected in 2016 by Nijmeijer et al. (2019).

Out of eleven potential reasons suggested, the most selected reason in favor of ECS for both the video group (53.1%) and text group (47.2%) was that they want to spare their child a life with a severe hereditary disorder (Table 3A). Out of thirteen suggested reasons, the most selected reason against ECS for both the video group (39.5%) and the text group (48.0%) was that nobody in the family has one of these disorders (Table 3B).

V Perceived personal consequences.

**TABLE 3:** Most important reasons in favor of and against ECS

	Text group^a^	Video group	*p*
	(*n* = 781)	(*n* = 789)	
	%	%	
A. Most important reasons in favor of ECS			
I want to spare my child from a life with a severe hereditary disorder	47.2	53.1	**.020**
I do not want a child with one of these 50 disorders	20.9	28.3	**.001**
I want to prepare myself for a child with one of these disorders	23.8	22.7	.597
A hereditary disorder occurs in my family	17.0	16.5	.769
I believe I have a great chance of being a carrier	16.6	13.2	.054
B. Most important reasons against ECS			
Nobody in the family has one of these disorders	48.0	39.5	**.001**
I am afraid of the test results	15.7	16.9	.552
I do not believe I have a great chance of being a carrier	20.4	14.8	**.004**
I do not believe I have a great chance of having a child with one of these 50 disorders	17.7	13.1	**.011**
I would not do anything with the results	12.9	12.8	.938

Note

Questionnaire part IV: the top 5 most frequently selected reasons in favor of and against ECS in order of frequency.

Significant differences *p* < .05 are presented in bold.

^a^ Data collected in 2016 by Nijmeijer et al. (2019).

There was a significant difference between respondents in the video group and text group regarding their agreement on most items of the domain ‘perceived personal consequences’ (Table 4). For example, respondents in the video group more often would consider in vitro fertilization (IVF) with embryo selection compared to the text group (respectively, 48.2% vs. 38.5%, *p* < .001).

**TABLE 4:** Statements regarding perceived personal consequences of ECS

	Text group^a^	Video group	*p*
	(*n* = 781)	(*n* = 789)	
	%	%	
A. Considerations regarding test results of ECS			
I would find it difficult if my child would be affected by one of the 50 disorders	76.6	82.8	**.002**
The results of a carrier test can help me in making decisions about having children	54.9	59.6	.063
If I do not participate, I am afraid I will regret it if my child is affected with one of the 50 disorders	49.0	56.3	**.004**
If I do not participate, I am afraid I will regret it later	42.9	51.7	**.000**
Offering a carrier test takes away the spontaneity of having children	43.9	42.1	.462
B. Perceived consequences as a carrier			
By preventing the birth of child with a severe hereditary disorder, a lot of suffering can be prevented	64.0	71.6	**.001**
It is important that the birth of a child with a severe hereditary disorder can be prevented	60.7	68.2	**.002**
If I were a carrier, I would find it difficult to inform my family members about their increased risk of being a carrier	37.9	34.6	.174
I am afraid people will look differently at me when they know I am a carrier	24.2	15.5	**.000**
C. Perceived reproductive choices as a carrier couple			
I find it important that carrier couples can prepare themselves for the birth of a child with a severe hereditary disorder	78.6	80.7	.297
I would consider an examination of the fetus during pregnancy (prenatal testing by chorionic villus sampling)	63.5	62.5	.674
As part of a carrier couple, I would consider in vitro fertilization (IVF) with embryo selection	38.5	48.2	**.000**
If my partner and I are a carrier couple, I would decide not to have (more) children	35.1	29.3	**.014**
I would take the risk and not take any action (the child is born as he or she is)	34.1	28.4	**.015**

Note

Questionnaire part V: agreement on statements.

Statistical differences *p* < .05 are presented in bold.

^a^ Data collected in 2016 by Nijmeijer et al. (2019).

## 4 DISCUSSION

The purpose of the current study was to assess the impact of an educational video compared to an educational text on the knowledge and attitudes toward preconception ECS for severe childhood onset disorders in the general population. MPS III was used as an example of the type of disorders that are included in ECS panels.

First, we show that genetic knowledge and the attitudes toward ECS, as assessed by our questionnaire, had not changed over 2 years' time. Therefore, we can assume that the difference in attitudes between the text group and video group is due to the mode of presenting the information and not to accumulating knowledge and awareness over time.

Watching the educational video led to an increased intended uptake of ECS testing. Moreover, respondents who watched the video had an overall more positive attitude toward preconception ECS compared to those who read the text. In addition, participants who were offered the video as source of information scored significantly higher on the genetic knowledge test. This is in line with earlier studies demonstrating the positive effect of using video to increase knowledge of (future) parents on Down Syndrome screening (Bjorklund et al., 2012; Hewison et al., 2001) and of members of a Jewish population on preconception carrier screening (Hardy et al., 2018). However, a study by Clayton et al. (1995) reported that written and video materials were equally effective in conveying information about preconception carrier screening for CF.

Surprisingly, almost 40% of the respondents who were offered the educational video still chose ‘absence of a genetic disorder in the family’ as most important reason not to take ECS, although the video extensively explained the concept of AR inheritance. Ong et al. (2018) also demonstrated that good genetic knowledge may not be sufficient to fully understand the core concepts of ECS, potentially compromising an informed decision. Nevertheless, this reason was less often chosen by the video group compared to the text group (49%), confirming that the video led to improved knowledge on the concept of AR inheritance.

Improved genetic knowledge in the video group does not necessarily mean this group would make a more informed decision compared to the text group or that the text group will make an uninformed decision. As the aim of ECS is to provide carrier couples with options for autonomous reproductive choices, it is also important to determine whether individuals make an *informed decision* to opt for screening or not, that is, a decision free of coercion and consistent with a persons' norms and values (Marteau, Dormandy, & Michie, 2001). To obtain information on the degree of informed choice, more insight is needed in the values people attach to potential benefits and harms of screening, the reasons why they (do not) opt for the test, and if this decision was in line with their own values and beliefs (Ames, Metcalfe, Dalton Archibald, Duncan, & Emery, 2015; Henneman et al., 2016).

This study also shows that participants who watched the video more often agreed that MPS III is a severe disease compared to those who read the educational text. The portrayal of real‐life images of MPS III patients in their home environment likely created more awareness on the possible impact of the disease. Previous research showed that explaining a disease by video increased the subjective understanding of that particular disease (Volandes et al., 2007). The fact that respondents who watched the video perceived MPS III as more severe may also explain the more favorable attitudes toward ECS in this group. Whereas several studies found that awareness of genetic diseases and the perceived severity of an affected child was positively associated with the intention to participate in carrier screening (Holtkamp et al., 2017; Ioannou et al., 2014; Voorwinden et al., 2017), Poppelaars et al. (2004) did not found such an association for CF screening. Although 84% of the participants in the video group perceived MPS III as severe, less than half of them indicated that they probably or certainly would participate in ECS. As was also shown in the study of Henneman et al. (2001), these results suggest that other (Health Belief Model related) factors may be more decisive. For example, participants might believe that ECS is not relevant for them due to the absence of a genetic disorder in the family or the outcome would not influence their reproductive decision‐making. Nevertheless, an illustration of the effect of more awareness about the potential impact of severe AR disorders included in screening panels is the remarkably high intended uptake rate of 68% in Western Australia (Ong et al., 2018) (compared to, e.g., 31% in the Netherlands (Nijmeijer et al., 2019)), as the uptake in Australia was assessed after substantial media coverage of parents who shared their personal story about their daughter with a severe genetic disorder and the importance of ECS (see, e.g*.,* https://science.anu.edu.au/news‐events/news/one‐small‐baby‐one‐giant‐leap‐genetic‐screening for more information). However, intended participation is not always a good reflection of the actual test uptake, since the actual test uptake may be influenced by other factors such as time and costs (Lakeman et al., 2009).

Another finding of our study was that all respondents perceived the actual risk of being a carrier or a carrier couple as high. Respondents in the video group perceived the risk of 1:600 of having a child with one of the 50 severe genetic disorders higher than the text group. The relation between risk perception and screening behavior is unclear, as some studies found that perceived risk was positively associated with intended participation (van der Pal, van Kesteren, van Wouwe, van Dommelen, & Detmar, 2013; Voorwinden et al., 2017), while others did not confirm this association (Holtkamp et al., 2017; Poppelaars et al., 2004).

It has been recognized that offering balanced information about genetic disorders and disabilities is important to facilitate informed choices in (prenatal) screening programs (Ahmed, Bryant, & Hewison, 2007; Williams, Alderson, & Farsides, 2002; Wright et al., 2015). In order to provide balanced information about MPS III in the current study, we included both visuals of MPS III patients in the first years of their life without disease characteristics as well as teenage patients completely dependent on care. In order to stay close to the script of the educational text, we did not include interviews with parents or other relatives in the video. This is in contrast with an earlier study assessing a web resource that provided information about disorders based on the testimonies of people with disabilities and their families to support choices in antenatal screening decisions (Ahmed et al., 2007). Although narratives would have balanced the information on the medical aspects of MPS III, it does not necessarily mean they are balanced in itself and may bias individuals' decision‐making in an unpredictable way (Bekker et al., 2013; Winterbottom, Bekker, Conner, & Mooney, 2008).

### 4.1 Study limitations

Some limitations of the current study need to be discussed. Firstly, the current study assessed attitudes toward a hypothetical test situation and therefore the outcomes may differ in a real world test situation. Secondly, we did not assess the level of genetic knowledge before offering the educational text or video. However, as no baseline differences in the familiarity with carrier screening and hereditary diseases were detected, we assume equal prior genetic knowledge between the text group and the video group. Secondly, we did not control whether participants actually read the educational text or fully watched the video, although we had emphasized its importance in the information at the start of the survey. Thirdly, we used MPS III as an example of the type of disorders included in ECS. As MPS III is a very severe progressive disorder, it may not be fully representative of all disorders included in ECS panels. However, we specifically took care not to show patients in the last stage of the disease, as we wanted to avoid emotional coercion. Fourthly, there was a difference in content between the educational text and the video, as in the video factual information on the risk of being a carrier couple (1 in 150) and the risk of having an affected child when being a carrier couple (1 in 600) was added. However, respondents in the text group were also informed about these risks as this was included in the questionnaire domain ‘perceived risk’. This may have minimized the influence of this difference in content. In addition, the video comprised additional information as the concept of AR inheritance was explained by animations and the example of the disorders included in the ECS panel, MPS III, was illustrated by video‐recordings of two patients. Therefore, the differences between the text group and video group might be caused by the different methods of presenting information, but also by the additional content.

### 4.2 Practice implications

As ECS is currently offered to individuals or couples without an a priori risk for, and limited awareness of the disorders included in the screening panels, it is important to educate the general public about the various aspect of ECS and the type of included disorders. This study shows that an educational online video may well be an effective supportive tool to this end and may be used by health care professionals, including genetic counselors and general practitioners.

### 4.3 Research recommendations

Although our educational video led to improved genetic knowledge compared to text, we show that surprising misconceptions about ECS may still remain. Further research may examine the effectiveness of different types and combinations of educational tools. This might include a combination of video and text, interactive web‐based learning, whether or not in combination with a brief contact with a trained counselor which might be done in person or by phone or video consultation. We believe that such studies are paramount to determine the optimal way to educate the general public on preconception ECS which is essential for responsible and effective introduction of this type of screening.

## 5 CONCLUSION

The results of our study are in line with the current era of online, visual and concise information retrieval as it shows that the use of video for educating the general public on preconception ECS and on the disorders generally included in such tests is superior to text and leads to increased genetic knowledge. Moreover, this study shows that potential uptake rates of ECS may increase with the use of video as educational tool. The success of preconception ECS programs should not be assessed merely on the uptake of screening but rather in terms of a measure of informed choice of (prospective) parents (Henneman et al., 2016). Online video may well be used as supportive tool to the pre‐test genetic counseling process to facilitate informed choice.

## AUTHOR CONTRIBUTIONS

Thirsa Conijn was involved in the conception and design of this work, analysis and interpretation of the data, and drafting the article. Stephanie Nijmeijer, Frits Wijburg, Phillis Lakeman, Lidewij Henneman, and Lotte Haverman were involved in the conception and design of the work, in analyses and interpretation of the data, and critically revising the manuscript for important intellectual content. Thirsa Conijn, Stephanie Nijmeijer, Frits Wijburg, and Lotte Haverman confirm that they had full access to all the data in the study and take responsibility for the integrity of the data and the accuracy of the data analysis. All of the authors gave final approval of this version to be published and agree to be accountable for all aspects of the work in ensuring that questions related to the accuracy or integrity of any part of the work are appropriately investigated and resolved.

## Compliance with ethical standards

### Conflict of interest

Thirsa Conijn, Stephanie Nijmeijer, Phillis Lakeman, Lidewij Henneman, Frits Wijburg, and Lotte Haverman are affiliated to a hospital that offers ECS in a non‐commercial setting.

### Human studies and informed consent

The Medical Ethics Committee of the Amsterdam UMC, the Netherlands, stated that Medical Research Involving Human Subject Act (WMO) does not apply to this study as it concerns an anonymous questionnaire study and therefore formal ethical approval was not necessary. Informed consent was obtained from participants prior to completing the online questionnaire.

### Animal studies

No non‐human animal studies were carried out by the authors for this article.

### Data Sharing and Data accessibility

The data that support the findings of this study are available from the corresponding author upon reasonable request.

## Supporting information

Appendix S1

Appendix S2

Appendix S3
