# Supplementary material for: Preconception expanded carrier screening: Impact of information presented by text or video on genetic knowledge and attitudes
Source: J Genet Couns. 2020 Sep 17;30(2):457–69. doi: 10.1002/jgc4.1332 (PMC8048558; doi:10.1002/jgc4.1332)
Supplement: Supplementary file 1 — Appendix S1 [file JGC4-30-457-s003.doc]

**Appendix S1**

**Supplementary material A.**

**Educational text**

Couples considering a (future) pregnancy can test their carrier status for 50 severe genetic disorders to identify if they have an increased risk of having a child with one of these disorders.

***What is a carrier?***

Everyone is a ‘carrier’ of one or more genetic disorders. When someone is a carrier of a certain disorder, he or she does not experience symptoms and will never have the disorder. Since these genetic disorders often do not occur in the family, people are not aware that they are carriers of this disorder. However, when both parents are carriers of **the same** genetic disorder, the couple has an increased risk (25%) of having an affected child in every pregnancy.

***What is a carrier test?***

A carrier test can identify whether someone is a carrier of a genetic disorder. For the carrier test, a blood sample is needed. The test does not provide a 100% certainty of having a healthy child.
- If the test results show that someone is ***not a carrier***, then there is still a very small risk of being a carrier of such genetic disorder.
- If the test results show that someone is a ***carrier***, than this is always 100% certain.

***When a couple are both carriers***Only when a couple are *both* carriers of **the same** disorder, there is an increased risk of having a child with a genetic disorder. In every pregnancy, there is a risk of 1 in 4 (25%) of having an affected child. Parents who are both carriers of the same disease have the following reproductive options:

- Accept the risk of a sick child.
- Examine during the pregnancy if the unborn child is affected (prenatal testing by chorionic villus sampling). If so, the couple can decide to terminate the pregnancy or to prepare for the birth of the child.
- Undergo in vitro fertilization (IVF). During this procedure, a healthy embryo is selected and placed in the womb (embryo selection).
- Make use of sperm or eggs from a donor who is not a carrier for the disorder.
- Pursue adoption.
- Decide not to have any (more) children.

***An example of a severe, untreatable, hereditary disorder is Sanfilippo disease.*** Sanfilippo disease is a metabolic disease with no available treatment. The disease is characterized by an increasing mental disability due to toxic waste that builds up in the entire body and the brain. The disease starts with a developmental delay around the age of 2 to 4 years. Subsequently, other abilities, such as speech, are lost. Later, problems with swallowing and mobility arise. Patients with Sanfilippo disease become completely care dependent and usually die before they reach adulthood.
