# Supplementary material for: Preconception expanded carrier screening: Impact of information presented by text or video on genetic knowledge and attitudes
Source: J Genet Couns. 2020 Sep 17;30(2):457–69. doi: 10.1002/jgc4.1332 (PMC8048558; doi:10.1002/jgc4.1332)
Supplement: Supplementary file 2 — Appendix S2 [file JGC4-30-457-s002.docx]

**Appendix S2**

**Supplementary material B.**

**Genetic knowledge test**

The following questions are meant to check if the information provided before the start of this questionnaire has been correctly understood. Choose one answer of which you think is the right one.

| **1.** | Even when a disease **does not occur in the family**, a couple can still have a child with one (or more) of these 50 diseases | - Correct - Incorrect - I do now know |
| --- | --- | --- |
| **2.** | **A carrier** of one of these 50 disorders can get health problems later in life | - Correct - Incorrect - I do now know |
| **3.** | **A carrier couple** has a chance of 1 in 4 (25%) in every pregnancy to get an affected child | - Correct - Incorrect - I do now know |
| **4.** | If **one partner** is carrier for one of the diseases, couples have an increased risk to have a child with that disease | - Correct - Incorrect - I do now know |
| **5.** | A carrier couple can **examine during pregnancy** if the unborn child is affected | - Correct - Incorrect - I do now know |
| **6.** | If the test results show that someone is not a carrier, then there is still a very small risk of being a carrier of such a genetic disorder. | - Correct - Incorrect - I do now know |
| **7.** | If the test results show that someone is not a carrier, a child can still be born with a different genetic disease | - Correct - Incorrect - I do now know |

**Perceived severity of MPS III disease**To what extent do you agree with the following statements? Tick the box that most closely represents your opinion.

|  | Strongly disagree | Disagree | Do not disagree / do not agree | Agree | Strongly agree |
| --- | --- | --- | --- | --- | --- |
| I believe that MPS III is a severe disease | □ | □ | □ | □ | □ |
| I believe that the life-expectancy of MPS III patients is very bad (severe patients often die before reaching adulthood) | □ | □ | □ | □ | □ |

**Perceived risk**The following questions are about the chances of having a child with one of the 50 hereditary diseases. Tick the box that most closely represents your opinion.

|  | Very low | Low | Not high / not low | High | Very high |
| --- | --- | --- | --- | --- | --- |
| How high do you consider a chance of 1:6 of being a carrier of a severe, hereditary disease | □ | □ | □ | □ | □ |
| How high do you consider a chance of 1:150 that both partners are carrier of the same severe hereditary disease? | □ | □ | □ | □ | □ |
| How high do you consider the chance of 1:600 per pregnancy of having a child with a severe, hereditary disease? | □ | □ | □ | □ | □ |
