# Supplementary material for: Preconception expanded carrier screening: Impact of information presented by text or video on genetic knowledge and attitudes
Source: J Genet Couns. 2020 Sep 17;30(2):457–69. doi: 10.1002/jgc4.1332 (PMC8048558; doi:10.1002/jgc4.1332)
Supplement: Supplementary file 3 — Appendix S3 [file JGC4-30-457-s001.doc]

**Appendix S3**

**Supplementary material C.**

**ECS questionnaire**

For the following questions, we are looking for your opinion. There is no right or wrong answer.

1. **Would you take a carrier test yourself?**

Tick the box that most closely represents your opinion.

| Would you take a carrier test yourself? | - Definitely - Probably - Maybe / maybe not - Probably not - Definitely not - I have taken a carrier test |
| --- | --- |

Why would / wouldn’t you take a carrier test?
…………………………………………………………………………………………………

…………………………………………………………………………………………………

1. **Your opinion towards the carrier test**

We would like to know what your thoughts and feelings are towards the carrier test. For the following questions, different words with opposite meanings are listed. Tick the box that most closely resembles your opinion. You can choose the middle box if your opinion is neutral.

***Example:***

*If you find ‘having children’ important, then tick the box on the right side.*

*If you find ‘having children’ less important, than tick a box that is more to the left side.*

*Tick one box for each word pair.*

|  | **Having children is….** | | | | | | |
| --- | --- | --- | --- | --- | --- | --- | --- |
|  | Unimportant | **□** | **□** | **□** | ***X*** | **□** | Important |
|  | Frightening | **□** | **□** | ***X*** | **□** | **□** | Nonfrightening |

| **That all couples considering a (future) pregnancy can take the carrier test for 50 severe hereditary disorders, I find …** | | | | | | |
| --- | --- | --- | --- | --- | --- | --- |
| Negative | **□** | **□** | **□** | **□** | **□** | Positive |
| Undesirable | **□** | **□** | **□** | **□** | **□** | Desirable |
| Frightening | **□** | **□** | **□** | **□** | **□** | Nonfrightening |
| Unwise | **□** | **□** | **□** | **□** | **□** | Wise |
| Nonreassuring | **□** | **□** | **□** | **□** | **□** | Reassuring |
| Unethical | **□** | **□** | **□** | **□** | **□** | Ethical |
| Illogical | **□** | **□** | **□** | **□** | **□** | Logical |
|  |  |  |  |  |  |  |
| **That I personally can take the carrier test for 50 severe hereditary disorders, I find…** | | | | | | |
| Negative | **□** | **□** | **□** | **□** | **□** | Positive |
| Undesirable | **□** | **□** | **□** | **□** | **□** | Desirable |
| Frightening | **□** | **□** | **□** | **□** | **□** | Nonfrightening |
| Unwise | **□** | **□** | **□** | **□** | **□** | Wise |
| Nonreassuring | **□** | **□** | **□** | **□** | **□** | Reassuring |
| Unethical | **□** | **□** | **□** | **□** | **□** | Ethical |
| Illogical | **□** | **□** | **□** | **□** | **□** | Logical |

1. **To what extent do you agree with the following statements?**

Tick the box that most closely represents your opinion.

|  |  | Strongly disagree | | Disagree | | Do not disagree / do not agree | | Agree | Strongly agree | |  |
| --- | --- | --- | --- | --- | --- | --- | --- | --- | --- | --- | --- |
|  | The carrier test can **avoid suffering** for **future** **parents** | □ | □ | | □ | | □ | | | □ | |
|  | Offering the carrier test **avoids much suffering** for the entire **family** | □ | □ | | □ | | □ | | | □ | |
|  | The carrier test can **prevent** **costs** for the **family** | □ | □ | | □ | | □ | | | □ | |
|  | The carrier test can **prevent** **costs** for the **society** | □ | □ | | □ | | □ | | | □ | |
|  | The carrier test should be **offered** to **every** **couple** that wants to have children | □ | □ | | □ | | □ | | | □ | |
|  | **Every** **couple** that wants to have children **must take** the carrier test | □ | □ | | □ | | □ | | | □ | |
|  | The carrier test creates **too** **high** **expectations** of the birth of a **healthy** **child** | □ | □ | | □ | | □ | | | □ | |
|  | The carrier test will be the first step in developing a **perfect** **child** | □ | □ | | □ | | □ | | | □ | |
|  | I am afraid of **discrimination** by **carriers** (for instance, by insurance companies and the social environment) | □ | □ | | □ | | □ | | | □ | |
|  | The **results** of a carrier test can help in choosing a partner | □ | □ | | □ | | □ | | | □ | |
|  | Offering a carrier test leads to **anxiety** | □ | □ | | □ | | □ | | | □ | |
|  | Offering the carrier test can cause people to **feel forced** to undergo testing | □ | □ | | □ | | □ | | | □ | |

1. **Your view on the carrier test**

The following questions are reasons to take or not to take the carrier test. A blood sample is needed for the carrier test.

|  | What would be the most important reason for you to **take** a carrier test?  *Select a maximum of 2 answers.* | - I believe I have a great chance of being a carrier - I believe I have a great chance of having a child with one of these 50 disorders - I want to spare my child from a life with a severe disorder - I do not want a child with one of these 50 disorders - Fear of regret if I do not take the test - I want to prepare myself for a child with one of these disorders - The wishes of my partner - A hereditary disorder occurs in my family - For my children (when they want to have children) - Advised by others, please specify …………… - Other, please specify …………………………. |
| --- | --- | --- |

|  | What would be the most important reason for you **not** **to** **take** a carrier test?  *Select a maximum of 2 answers.* | - Nobody in the family has one of these disorders - I do not believe I have a great chance of being a carrier - I do not believe I have a great chance of having a child with one of these 50 disorders - I have taken a carrier test with only the disorder for which I have an increased risk - I do not believe the disorders are severe enough - I would not do anything with the results; I believe I can handle a sick child well - I am / my partner is (too far along) pregnant - I am afraid of the test results - My partner does not want to take the test - The price of the test - Other, please specify ………………………. |
| --- | --- | --- |

1. **To what extent do you agree with the following statements?**

Suppose you would **consider** the **carrier** **test** before a pregnancy. To what extent do you agree with the following statements?

|  | |  | Strongly disagree | | Disagree | | Do not disagree / do not agree | | Agree | Strongly agree | |  |
| --- | --- | --- | --- | --- | --- | --- | --- | --- | --- | --- | --- | --- |
| **1.** | If I do not participate, I am **afraid** I will **regret** it later | | □ | □ | | □ | | □ | | | □ | |
| **2.** | If I do not participate, I am afraid I will feel **guilty** if my child is affected by one of the 50 disorders | | □ | □ | | □ | | □ | | | □ | |
| **3.** | The results of the carrier test can **help** me **in making decisions** about having children | | □ | □ | | □ | | □ | | | □ | |
| **4.** | I would find it **a burden** if my child would be affected with one of the 50 disorders | | □ | □ | | □ | | □ | | | □ | |
| **5.** | Offering the carrier test takes away the **spontaneity** of having children | | □ | □ | | □ | | □ | | | □ | |

Suppose you and your partner are both carriers of the same severe genetic disorder and you therefore have an increased risk (25%) of having a child with a severe genetic disorder. To what extent do you agree with the following statements?

|  | Strongly disagree | Disagree | Do not disagree / do not agree | Agree | Strongly agree |
| --- | --- | --- | --- | --- | --- |

| **1.** | I am afraid people will look differently at me when they know I am a **carrier** | □ | □ | □ | □ | □ |
| --- | --- | --- | --- | --- | --- | --- |
| **2.** | If I were a carrier, I would find it difficult to inform my **family members** about  their **increased risk** | □ | □ | □ | □ | □ |
| **3.** | It is important that the **birth** of a child with a severe hereditary disorder can be **prevented** | □ | □ | □ | □ | □ |
| **4.** | By **preventing** the **birth** of child with a severe hereditary disorder, a lot of **suffering** can be **prevented** | □ | □ | □ | □ | □ |

Suppose you and your (future) partner are a **carrier** **couple** for a severe hereditary disorder and you are **pregnant**. To what extent do you agree with the following statements?

|  | |  | | Strongly disagree | | Disagree | | Do not disagree / do not agree | | Agree | Strongly agree | |  |
| --- | --- | --- | --- | --- | --- | --- | --- | --- | --- | --- | --- | --- | --- |
| **1.** | I would take the risk and **not take any action** (the child will be born as he or she is) | | □ | | □ | | □ | | □ | | | □ | |
| **2.** | If my partner and I are a carrier couple, I would decide **not** to have (more) children | | □ | | □ | | □ | | □ | | | □ | |
| **3.** | I would consider **examination** of the fetus **during the pregnancy** (prenatal testing by chorionic villus sampling) | | □ | | □ | | □ | | □ | | | □ | |
| **4.** | As part of a carrier couple, I would **consider** **in vitro fertilization (IVF)** with embryo selection | | □ | | □ | | □ | | □ | | | □ | |
| **5.** | I find it important that carrier couples can **prepare** **themselves** for the birth of a child with a severe hereditary disorder | | □ | | □ | | □ | | □ | | | □ | |
| **6.** | I would **consider termination of the pregnancy** if the child is affected with one of the 50 disorders | | □ | | □ | | □ | | □ | | | □ | |
| **7.** | I am **against** **abortion** of a child with one of these 50 disorders | | □ | | □ | | □ | | □ | | | □ | |

General questions

| What is your marital status? | - Single - In a relationship - Married / living together / registered partners - Divorced - Widow(er) - Other, ……………………. |
| --- | --- |
|  |  |
| Do you have any religious beliefs? | - Yes - No - I do not know - I do not want to say |

| What is your family composition? | - I do not have any children, and I do not want children - I do not have any children, but I would like to have children - I do not have any children but I am (my partner is) currently pregnant - I would have liked to have children, but I have remained childless - I have children - I do not want any more children - I would like to have more children - I have children and I am (my partner is) currently pregnant - I am uncertain whether I would like to have children - Other ……………………. |
| --- | --- |

| Have you ever heard of a carrier test before this questionnaire? | - No - Yes   - Via family   - Via friends / colleagues   - Via a general practitioner   - Via a midwife   - Via television / radio   - Via a newspaper   - Via the internet   - I do not remember how   - Other, ………….. |
| --- | --- |

| Have you ever taken a carrier test? | - No - Yes, for the following disease: ……………………. |
| --- | --- |
|  |  |
| Do you (or have you) know(n) someone with a hereditary disorder? | - No - Yes, a family member:   - Which family member: ……………………   - Which disorder: …………………………… - Yes, someone else:   - Which person: …………………………......   - Which disorder: …………………………… |

Do you have any comments you would like to share with us?

…………………………………………………………………………………………………

…………………………………………………………………………………………………

**Thank you for completing the questionnaire.**
